# Association of preoperative seizures with tumor metabolites quantified by magnetic resonance spectroscopy in gliomas

**DOI:** 10.1038/s41598-021-86487-6

**Published:** 2021-04-12

**Authors:** Shunsuke Nakae, Masanobu Kumon, Kazuhiro Murayama, Shigeo Ohba, Hikaru Sasaki, Joji Inamasu, Kiyonori Kuwahara, Seiji Yamada, Masato Abe, Yuichi Hirose

**Affiliations:** 1 Department of Neurosurgery, Fujita Health University, 1-98 Dengakugakubo, Kutsukake-cho, Toyoake, Aichi 470-1192 Japan; 2 Department of Radiology, Fujita Health University, Toyoake, Aichi Japan; 3 Department of Neurosurgery, Keio University, Tokyo, Japan; 4 Department of Pathology, Fujita Health University, Toyoake, Aichi Japan

**Keywords:** Cancer, Neuroscience

## Abstract

Seizures are common in patients with gliomas; however, the mechanisms of epileptogenesis in gliomas have not been fully understood. This study hypothesized that analyzing quantified metabolites using magnetic resonance spectroscopy (MRS) might provide novel insights to better understand the epileptogenesis in gliomas, and specific metabolites might be indicators of preoperative seizures in gliomas. We retrospectively investigated patient information (gender, age at diagnosis of tumor, their survival time) and tumor information (location, histology, genetic features, and metabolites according to MRS) in patients with gliomas. The data were correlated with the incidence of seizure and analyzed statistically. Of 146 adult supratentorial gliomas, *isocitrate dehydrogenase* (*IDH*) mutant tumors significantly indicated higher incidence of preoperative seizures than *IDH* wild-type gliomas. However, MRS study indicated that glutamate concentration in *IDH* wild-type gliomas was higher than that in *IDH* mutant gliomas. Glutamate was not associated with high frequency of preoperative seizures in patients with gliomas. Instead, increased total *N*-acetyl-l-aspartate (tNAA) was significantly associated with them. Moreover, multivariable analysis indicated that increased level of tNAA was an independent predictor of preoperative seizures. According to MRS analysis, tNAA, rather than glutamate, might be a useful to detect preoperative seizures in patient with supratentorial gliomas.

## Introduction

Seizures are common in patients with brain tumor. Although the incidence of seizures is estimated to be about 30% in brain tumors, it greatly differs depending on its histology^1–3^. In gliomas, seizures in patients with low-grade gliomas are more frequent than those in patients with high-grade gliomas (HGGs)^1, 4, 5^. After the finding of *isocitrate dehydrogenase* (*IDH*) *1/2* mutations^6, 7^, gliomas harboring mutant *IDH* and wild-type *IDH* have been considered to be distinct tumors that derived from different linkages because these two genetic subtypes have totally different characteristics including survival time, mean age at diagnosis, and tumor location^8–10^. Recently, reports indicating the association between seizures and *IDH* mutation status have increased, suggesting that its mutation is associated with high seizure frequency^5, 11–13^.

Although the mechanisms of presenting seizures are not fully understood, previous studies described some possible mechanisms for epileptogenesis in gliomas. Uptake of glutamate decreases in glioma cells owning to the lack of sodium-dependent excitatory amino acid transporters 1 and 2^14, 15^, whereas glutamate release from glioma cells is promoted by the system Xc-cystine/glutamate transporter^16^. These events result in a high concentration of extracellular glutamate and consequently induce seizure activities in gliomas^16^. In addition, previous studies reported that *IDH* mutation leads to the accumulation of D-2-hydroxyglutarate (2HG), which is structurally similar to glutamate, suggesting that 2HG acts as a glutamate receptor agonist, thus, resulting in high seizure frequency in *IDH* mutant gliomas^5, 15^. Moreover, signals for activating gamma-aminobutyric acid (GABA), which is known as an inhibitory transmitter, also weaken in gliomas^17^.

Magnetic resonance spectroscopy (MRS) quantifies levels of metabolites and some metabolites are associated with seizure activity^18^. We hypothesized that the analysis of tumor metabolites using MRS might provide novel insights for epileptogenesis in gliomas. Moreover, because varied symptoms are observed in patients with seizures especially in cases of focal aware seizures (FAS), such as auditory, olfactory, or autonomic seizures, it is helpful in the detection of seizures if MRS analysis indicates that some metabolites are associated with seizure frequency in patients with gliomas. Therefore, this study retrospectively investigated the relationship between preoperative seizure frequency in patients with glioma and tumor characteristics such as genetic information and tumor metabolites.

## Results

### Characteristics and seizure frequency of patients with glioma

A total of 163 patients were diagnosed as supratentorial gliomas between 2014 and 2019. They were histologically classified into four groups based on the World Health Organization (WHO) classification 2016: diffuse astrocytic and oligodendroglial tumors, other astrocytic tumors, ependymal tumors, and neuronal and mixed neuronal-glial tumors. In 163 patients, 146 were categorized into diffuse astrocytic and oligodendroglial tumors, 11 into other astrocytic tumors, 4 in ependymal tumors, 2 in neuronal and mixed neuronal-glial tumors. Because the other three histological groups account for 10% in supratentorial gliomas, further analyses were focused on patients with diffuse astrocytic and oligodendroglial tumors.

Among the 146 patients, 81 experienced seizures during their clinical courses, whereas 50 experienced seizure preoperatively. Of the 54 patients with preoperative seizures, 15 were seizure free after surgery. 43 of 146 patients were administered two or more AEDs in their clinical courses, and 37 of 136 patients were classified as Engel class III or IV after the initial surgery, suggesting that less than 30% of patients with diffuse gliomas were considered to have drug-resistant epilepsy according to this study. The mean age of patients with preoperative seizures was 47.6 years, whereas that of patients without preoperative seizures was 61.4 years (*P* < 0.001). These characteristics are presented in Table 1. AEDs were administered in 125 patients in their clinical courses for prevention or suppression of seizures: 1 AED in 92 patients, 2 AEDs in 38 patients, and 3 AEDs in 7 patients. The most frequently used AED was levetiracetam (N = 61.6%), followed by perampanel (10.3%), lacosamide (9.6%), valproate (9.6%), lamotrigine (5.5%), carbamazepine (4.1%), zonisamide (1.4%), phenobarbital (0.7%), and phenytoin (0.7%).

**Table 1:** Characteristics of patients with gliomas and the incidence of preoperative seizure.

Total diffuse astrocytic and oligodendroglial tumors (N = 146)		*P* values
**Mean age at diagnosis (range)**	56.7 years (22–89 years)	
Mean age of patients with preoperative seizures	47.6 years	** < 0.001**
Mean age of patients without preoperative seizures	61.4 years	
**Sex; %, female**	45.9%	
Preoperative seizure frequency of male patients	40.5%	0.12
Preoperative seizure frequency of female patients	26.8%	
**Laterality; %, left**	52.2%	
Preoperative seizure frequency of right-side tumors	25.0%	**0.02**
Preoperative seizure frequency of left-side tumors	45.7%	
**WHO grade; %, grade II, III, and IV, respectively**	27.4, 18.5, and 54.1%	
Preoperative seizure frequency of WHO grade II tumors	57.1%	0.42*
Preoperative seizure frequency of WHO grade III tumors	46.2%	0.02**
Preoperative seizure frequency of WHO grade IV tumors	17.9%	

bold values indicate in p <  0.05 and consequently two analyzed categories were considered to be significantly different.

*IDH, isocitrate dehydrogenase*; *WHO* World Health Organization.

*Comparison of preoperative seizure frequency between WHO grade II and III tumors.

**Comparison of preoperative seizure frequency between WHO grade III and IV tumors.

### *IDH* wild-type gliomas in diffuse astrocytic and oligodendroglial tumors

Because *IDH* wild-type and mutant gliomas have totally distinct characteristics, we evaluated the incidence of seizures for each genetic subtype separately. Consequently, 92 patients with diffuse astrocytic and oligodendroglial tumors harbored wild-type *IDH*. Of this genetic subgroup, 22 patients experienced seizures preoperatively, and 17 patients were classified as Engel class III or IV. The mean age of patients with preoperative seizures was significantly higher than that of patients without preoperative seizures (*P* = 0.01), and WHO grade of patients with preoperative seizures were significantly lower than that of patients without preoperative seizures (*P* = 0.03) (Table 2).

**Table 2:** Characteristics of patients with gliomas and incidence of preoperative seizure in each genetic subtype.

		*P* values
**IDH wild-type gliomas in diffuse astrocytic and oligodendroglial tumors (N = 92)**		
Mean age at diagnosis (range)	65.8 years (32–89 years)	
Mean age of patients with preoperative seizures	60.4 years	**0.02**
Mean age of patients without preoperative seizures	67.5 years	
Sex; %, female	41.3%	
Preoperative seizure frequency of male patients	25.9%	0.77
Preoperative seizure frequency of female patients	21.1%	
Laterality (%, left)	44.6%	
Preoperative seizure frequency of right-side tumors	17.3%	0.18
Preoperative seizure frequency of left-side tumors	44.4%	
WHO grade; %, WHO grade IV	82.6%	
Preoperative seizure frequency of WHO grade II or III tumors	50.0%	**0.02**
Preoperative seizure frequency of WHO grade IV tumors	18.4%	
**IDH mutant gliomas in diffuse astrocytic and oligodendroglial tumors (N = 54)**		
Mean age at diagnosis (range)	41.2 years (22–72 years)	
Mean age of patients with preoperative seizures	37.6 years	**0.01**
Mean age of patients without preoperative seizures	45.1 years	
Sex; %, female	53.7%	
Preoperative seizure frequency of male patients	68.0%	**0.03**
Preoperative seizure frequency of female patients	34.5%	
Laterality; %, left	64.7%	
Preoperative seizure frequency of right-side tumors	44.4%	0.41
Preoperative seizure frequency of left-side tumors	60.6%	
WHO grade; %, WHO grade II	70.4%	
Preoperative seizure frequency of WHO grade II tumors	55.3%	0.64
Preoperative seizure frequency of WHO grade III or IV tumors	43.8%	
Histology; %, oligodendrogliomas	44.4%	
Preoperative seizure frequency of oligodendrogliomas	54.2%	0.98
Preoperative seizure frequency of astrocytomas	50.0%	

bold values indicate in p <  0.05 and consequently two analyzed categories were considered to be significantly different.

*IDH, isocitrate dehydrogenase*; *WHO* World Health Organization.

### *IDH* mutant gliomas in diffuse astrocytic and oligodendroglial tumors

In this study, 54 patients were categorized into *IDH* mutant gliomas. 28 patients experienced seizures preoperatively, and 20 patients were classified as Engel class III or IV. The mean age of patients with preoperative seizures was significantly higher than that of patients without preoperative seizures (*P* = 0.01), and the frequency of preoperative seizure in male patients was significantly higher than that in female patients (*P* = 0.03). High-grade tumors in this genetic subtype indicated a lower frequency of preoperative seizures than low-grade tumors although the difference was not significant. Regarding genetic features, 24 of 54 patients harbored 1p/19q codeletions, i.e., oligodendroglial tumors, and 25 patients harbored *TP53* mutations. We compared the difference in seizure frequency between *IDH* mutant gliomas harboring 1p/19q codeletions and those without 1p/19q codeletions, and the difference was not statistically significant (Table 2). Preoperative seizure frequency of tumors harboring *TP53* mutations was lower than that of tumors harboring wild-type *TP53* (48.0% vs 55.2%, *P* = 0.80). We compared seizures in the entire clinical courses, preoperative seizures, and seizure prognoses between these two genetic subtypes. Consequently, patients with *IDH* mutant gliomas indicated a higher seizure frequency and worse seizure prognosis than those with *IDH* wild-type gliomas (Table 3).

**Table 3:** Comparisons between *IDH* mutant and wild-type gliomas.

	*IDH* mutant	*IDH* wild-type	*P* value
Seizure frequency	40 (74.1%)	41 (44.6%)	**0.001**
Postoperative seizure frequency	28 (51.9%)	23 (25.0%)	**0.002**
Engel class III or IV	19 (35.2%)	17 (18.5%)	**0.04**
Glutamate concentration based on MRS	3.07	4.01	**0.004**
tNAA concentration based on MRS	3.03	2.53	0.33

bold values indicate in p <  0.05 and consequently two analyzed categories were considered to be significantly different.

*IDH isocitrate dehydrogenase*; *tNAA* total *N*-acetyl-l-aspartate.

### Overall survival and its association with preoperative seizure frequency

**Figure 1:**
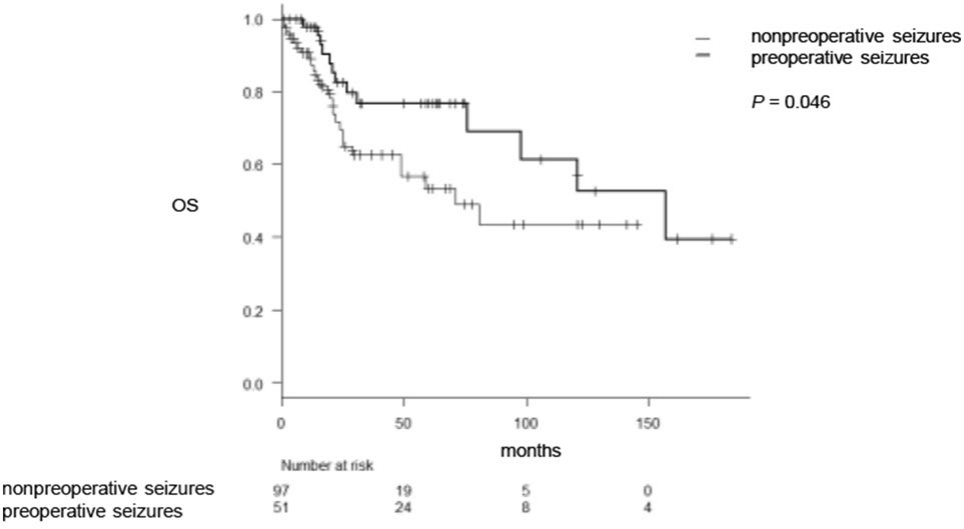
Comparison of survival between patients with preoperative and nonpreoperative seizures in *IDH* mutant and wild-type gliomas. OS, overall survival.

We discussed the association between survival in supratentorial gliomas and preoperative seizure frequency. Consequently, a Kaplan–Meier curve indicated that patients with preoperative seizures have a significantly better prognosis than those without preoperative seizures (*P* = 0.046) (Fig. 1). Multivariable analysis indicated that a preoperative seizure was not an independent prognostic factor in diffuse astrocytic and oligodendroglial tumors (*P* = 0.57), and high-grade tumor (WHO grade III or IV) and wild-type *IDH* were negative prognostic factors for patient survival (*P* = 0.002 and *P* = 0.014, respectively). We also analyzed the correlation between OS and preoperative seizures in *IDH* wild-type gliomas, indicating that there were no significant prognostic differences between patients with and without preoperative seizures (31 vs 25 months).

### Quantification of metabolites in MRS and its association with preoperative seizure frequency

Of examined tumor metabolites, GABA and 2HG were excluded from this study because %SD values of them were greater than 30% in most cases. We compared the quantified values of glutamate and total *N*-acetyl-l-aspartate (tNAA) between *IDH* mutant and wild-type gliomas. Consequently, glutamate concentration in *IDH* wild-type gliomas was significantly higher than that in *IDH* mutant gliomas. In contrast, tNAA concentration, representing density of normal neuron, was higher in *IDH* mutant gliomas than that in *IDH* wild-type gliomas (Table 3).

We first analyzed glutamate concentration together with both genetic subtypes, indicating that glutamate concentration was almost the same in the comparison of preoperative seizure vs nonpreoperative seizure (Table 4). We assumed that these results were caused by the findings that extracellular glutamate concentration in *IDH* wild-type gliomas was significantly higher than that in *IDH* mutant gliomas, whereas seizure frequency in *IDH* mutant gliomas was significantly higher than that in *IDH* wild-type gliomas. We then investigated glutamate for each genetic subtype separately. However, the differences in both genetic subtypes were not significant (Table 4).

**Table 4:** Glutamate and tNAA quantification based on MRS and its association with preoperative seizures.

	Glutamate	*P* value	tNAA	*P* value
**Total gliomas**				
Preoperative seizures	3.67 (25)	0.91	3.52 (30)	**0.008**
Without preoperative seizures	3.71 (42)		2.13 (39)	
***IDH wilt-type gliomas***				
Preoperative seizures	4.30 (11)	0.51	3.70 (13)	0.06
Without preoperative seizures	3.91 (33)		1.99 (28)	
***IDH mutant gliomas***				
Preoperative seizures	3.18 (14)	0.47	3.38 (17)	0.24
Without preoperative seizures	2.87 (9)		2.47 (11)	

bold values indicate in p <  0.05 and consequently two analyzed categories were considered to be significantly different.

*IDH, isocitrate dehydrogenase*; *tNAA* total *N*-acetyl-l-aspartate.

The numbers in the parenthesis indicate the number of analyzed cases patients who fulfilled the inclusion criteria based on %SD values.

We next investigated tNAA values in diffuse astrocytic and oligodendroglial tumors. Consequently, tNAA concentration in patients with preoperative seizures was significantly higher than that in patients without preoperative seizures (*P* = 0.008). Each genetic subtype indicated similar results, although the difference was not statistically significant (Table 4). Comprehensive data of gliomas including WHO grade, *IDH* mutation status, and quantified tumor metabolites, were summarized in Table 5. ROC curve using tNAA for preoperative seizures revealed that AUC was 0.70 and the cut-off value was 2.65 for differentiating preoperative seizures from nonpreoperative seizures. Because low-grade tumor, mutant *IDH*, and tNAA quantified by MRS were significantly associated with preoperative seizure frequency according to univariable analysis, we conducted multivariable analysis comparing these three factors. Consequently, it indicated that increased concentration of tNAA was an independent predictor of preoperative seizures (*P* = 0.03) (Table 6).

**Table 5:** Comprehensive data of gliomas in this study including WHO grade, *IDH* mutation status, and quantified mean values of glutamate and tNAA based on MRS.

	Preoperative seizures	Mutant IDH	Glutamate	tNAA
WHO grade II (42)	57.1%	90.5%	3.18 (18)	3.51 (24)
WHO grade III (26)	46.2%	57.7%	3.69 (11)	2.74 (11)
WHO grade IV (78)	17.9%	1.3%	3.92 (37)	1.91 (33)

*WHO* World Health Organization; *IDH, isocitrate dehydrogenase*; *tNAA* total *N*-acetyl-l-aspartate.

The numbers in the parenthesis indicate the number of analyzed cases.

**Table 6:** Multivariable analysis of factors associated with preoperative seizures in gliomas.

Variables	OR	95% CI	*P* value
WHO grade III or IV	0.66	0.13–3.22	0.60
Mutant *IDH*	2.94	0.65–13.40	0.16
tNAA (> 2.65)	3.66	1.17–11.50	**0.03**

*WHO* World Health Organization; *IDH isocitrate dehydrogenase*; *tNAA* total *N*-acetyl-l-aspartate.

## Discussion

We retrospectively investigated correlations between preoperative seizure frequency and various factors such as patient clinical information, genetic subtype, and metabolites quantified by MRS. Consequently, we clarified that increased tNAA is significantly associated with preoperative seizures in patients with gliomas. To the best of our knowledge, this is the first study to investigate factors associated with the incidence of seizures including genetic information and tumor metabolites comprehensively.

In this study, patients with preoperative seizures were significantly younger than those without preoperative seizures in supratentorial gliomas. In addition, WHO grade of patients with preoperative seizures was significantly lower than that of patients without preoperative seizures. A survival curve indicated that patients with preoperative seizures indicated significantly longer survival than those with nonpreoperative seizures. Because *IDH* wild-type and mutant gliomas have distinct characteristics, we investigated seizure frequency in these genetic subtypes separately. In *IDH* wild-type gliomas, elderly patients and high-grade tumors were significantly associated with low frequency of preoperative seizures as well. In *IDH* mutant gliomas, patients with preoperative seizures were significantly younger than those without preoperative seizures. Low-grade tumors showed higher frequency of preoperative seizures than high-grade tumors, whereas genetic information, such as 1p/19q co-deletions and *TP53* mutations, was not associated with high seizure frequency.

It is known that increased levels of extracellular glutamate are associated with high frequency of seizures, and extracellular glutamate concentration is increased in gliomas^16^. However, in MRS study, a high concentration of glutamate seems to be less associated with high frequency of preoperative seizures because glutamate concentration in *IDH* wild-type gliomas was significantly higher than that in *IDH* mutant gliomas, whereas seizure frequency in *IDH* mutant gliomas was much higher than that in *IDH* wild-type gliomas. A higher concentration of glutamate in *IDH* mutant gliomas was previously found in another MRS study and another metabolite analysis^19, 20^. These results suggest that only increased levels of extracellular glutamate are not enough for explanation of high frequency of seizures in gliomas. On the other hand, previous studies reported that glutamate binds to its receptor, a-amino-3-hydroxy-5-methylisoxazole-4-propionic acid (AMPA) receptors and promotes cell proliferation in tumor cells^21, 22^, suggesting that less amounts of glutamate may have more opportunities to bind to its receptors on neuronal tissues in high-grade tumors. Therefore, we hypothesized that the high density of normal neuron in tumoral tissues may be associated with high frequency of preoperative seizures in gliomas, and thus investigated concentration of tNAA, a marker for the density of neuronal normal tissues in the brain^23^. As expected, tNAA was significantly higher in a preoperative seizure group than in a nonpreoperative seizure group. Moreover, multivariable analysis indicated that increased tNAA is an independent predictor of preoperative seizures. In this study, patients with preoperative seizures were significantly younger than those with nonpreoperative seizures. This finding might support the correlation of high frequency of seizures with increased levels of tNAA because a previous study reported that tNAA is decreased with age in healthy human subjects^24^.

Unfortunately, we could not examine the effects of 2HG on epileptic seizures because the quantification of 2HG by MRS was unsuccessful. Another MRS study confirmed that 2HG concentration in *IDH* mutant gliomas was significantly higher than those in *IDH* wild-type gliomas^19^. Therefore, if 2HG prompts seizure activity, high frequency of seizures in *IDH* mutant gliomas might be reasonable because *IDH* mutation causes 2HG accumulation. However, of the ionotropic glutamate receptors, 2HG binds to N-methyl-D-aspartate (NMDA) receptors and does not bind to AMPA receptors in a human artificial model^5, 25^. It is still unclear whether the NMDA receptor is associated with epileptogenesis. In rodent models, NMDA, AMPA, and kainite have been reported to promote seizures^26, 27^. In contrast, NMDA-induced seizures in a rodent model are not well characterized, and the equivalent human seizure type is not well understood^28^. Taken together, we supposed that, although HGGs generate high concentrations of extracellular glutamate, much of these binds to AMPA receptors in tumor cells, causing further cell proliferation, and a little amount of these bind to AMPA receptors in neuronal tissues, causing low seizure frequency.

This study has certain limitations. First, it is difficult to precisely evaluate preoperative seizures. We believe that evaluation based on preoperative seizures is desirable to discuss the correlation between gliomas and the incidences of seizures, because seizures in the entire clinical courses include postoperative seizures that may result from surgical manipulations. Moreover, the postoperative follow-up periods varied, depending on the patient’s survival time. Therefore, we focused on the preoperative seizures in this study, although seizures, especially in patients with FAS, manifest as various symptoms, making the diagnosis of preoperative seizures difficult. Moreover, the seizures were assessed based on the medical record of each patient, and a diagnosis of epilepsy was established as per the judgement of the attending neurosurgeons for glioma patients. Unfortunately, in most cases, electroencephalography was not performed. This might have led to the underestimation of the prevalence of preoperative seizures, especially in patients with *IDH* mutant gliomas. Second, in this study, quantification of GABA and 2HG was unsuccessful. A previous study stated that long TE (TE = 97 ms) could provide reliable values of glutamate, glutamine, GABA, and 2HG^29^. Moreover, we used similar conditions (TE = 95 ms) to quantify these molecules; however, the %SD values did not meet our inclusion criteria. Another study reported that the concentration of tNAA and glutamate could positively affect the 2HG values when short TE was used^30^, suggesting that accurate quantification of these molecules is challenging. Technical issues involved in the detection of these molecules with MRS remain a challenge; however, this problem is expected to be resolved in the future.

In conclusion, we retrospectively investigated the association between the incidence of preoperative seizure and various factors, such as patient backgrounds, histology, genetic information, and metabolites quantified by MRS. Accordingly, MRS analysis indicated that glutamate concentration in *IDH* wild-type gliomas was higher than that in *IDH* mutant gliomas although *IDH* mutant gliomas significantly showed higher frequency of preoperative seizures than *IDH* wild-type gliomas. Instead, increased concentration of tNAA was an independent predictor of preoperative seizures in patients with supratentorial gliomas. This finding suggests that tNAA might be informative for the detection of preoperative seizures in patients with glioma, although the diagnosis of epileptic seizures should be made considering their symptoms, tumor location, and electroencephalographical patterns.

## Methods

### Patients

Patients who were histologically diagnosed as having supratentorial gliomas in the Department of Neurosurgery, Fujita Health University, between 2014 and 2019 were included. Resected tumor tissues were diagnosed by neuropathologists according to WHO classification 2016. Tumor characteristics were examined by magnetic resonance imaging (MRI) preoperatively. Resected tumors were diagnosed by neuropathologists. Age at diagnosis, tumor location, incidence of seizures, quantified metabolites according to MRS, extent of tumor resection, histological diagnosis, molecular information, postoperative therapy, overall survival (OS), seizure prognosis and use of antiepileptic drugs (AEDs) were retrospectively investigated. Information about seizures was obtained from the medical records of the patients with gliomas. The preoperative seizure rate was calculated. Seizure prognoses were evaluated as per Engel classification only patients where the follow-up time was > 1 year postoperatively.

### Genetic analysis

For resected tumors, chromosomal copy number and mutation status of specific genes, such as *IDH1/2* and *TP53*, were analyzed. Copy number and mutation analyses were performed by metaphase CGH and the Sanger method, respectively. Before genetic analyses, written informed consent was obtained from each patient. The extracted DNA was basically used from freshly frozen tissue for direct sequencing (FFPE samples were used if not available) and from FFPE for CGH analysis. These procedures were described in our previous studies^10, 31, 32^. In some cases, sufficient quantity of DNA was not available for direct sequencing. In such cases, we used immunohistochemistry for these mutation analyses.

### Magnetic resonance spectroscopy

**Figure 2:**
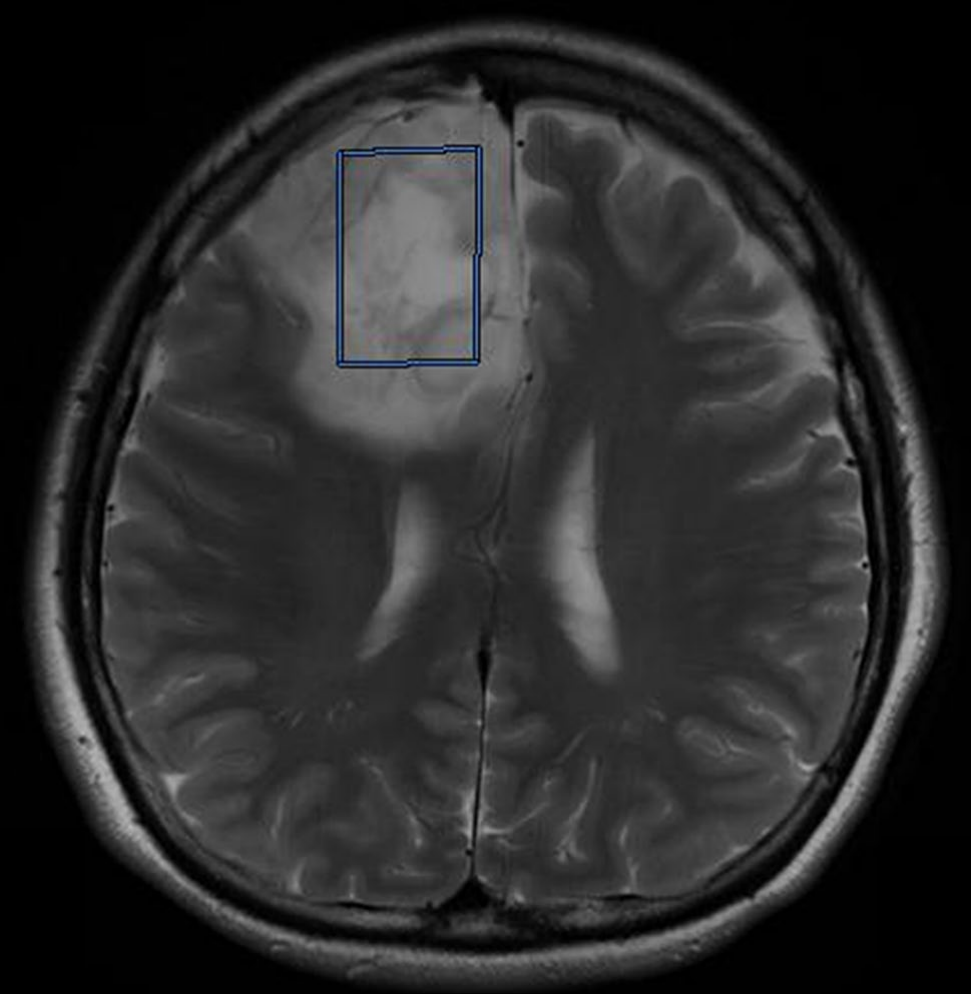
An MRI image showing an example of setting the voxel of interest in this study.

MRI system with a 32-channel head coil (Vantage Titan 3 T; Canon Medical Systems Corporation, Otawara, Japan) was used in this study. A single-voxel ^1^H-MRS with a point-resolved spectroscopy sequence was performed using the following standard parameters: TR/TE 2000 ms/144 ms and 2000 ms/35 ms, flip angle 90°, voxel size 15 × 15 × 15 mm (standard protocol, depending on the size and shape of the lesion), bandwidth 1.27 Hz/point, NEX 128. The voxel of interests (VOIs) were decided on the three-dimensional T2-weighted images by a single neuroradiologist to minimize the variation of the VOI location as per the following rule: the maximum size of VOI was set within a tumor not to include its border (Fig. 2). Tumor metabolites were examined as follows: GABA, glutamate, tNAA, and 2HG. The Analyzed echo time was based on previous studies^33, 34^. LCModel (Stephen Provencher, Oakville, Ontario, Canada) was used to automatically analyze all spectra data^35^. The obtained data were analyzed based on the Cramer-Rao lower bound with %SD value, and the quantified values of these metabolites were considered as reliable if their %SD values were 30 or less^19, 36^.

### Statistical analysis

The association of seizures with each characteristic was analyzed using a Fisher’s exact test for categorical variables and Student’s *t*-test for continuous variables. The data distribution was examined before analyses of Student’s *t*-test. Survival between the two groups was analyzed using Kaplan–Meier method and Cox log-rank test for comparison. Cox proportional hazards models were used to obtain the multivariable hazard ratio to clarify the prognostic factors in patients with glioma. Logistic regression analysis was performed to clarify the factors associated with preoperative seizures. Receiver operating characteristic (ROC) curve was analyzed, and the area under the curve (AUC) was calculated. The EZR software was used for all data analyses. *P* < 0.05 was considered statistically significant.

### Ethics declarations

This study was approved by the ethical committee at the Fujita Health University (CI20-125) and confirmed to the principles of the Declaration of Helsinki. Written informed consents were obtained from all patients for genetic analyses.
